# Sorafenib as a second-line treatment in metastatic renal cell carcinoma in Mexico: a prospective cohort study

**DOI:** 10.1186/s12885-020-07720-5

**Published:** 2021-01-05

**Authors:** Ana Elena Martín-Aguilar, Haidé Núñez-López, Juan C. Ramirez-Sandoval

**Affiliations:** 1 Centro Médico Nacional Siglo XXI, Av. Cuauhtémoc 330, Doctores, Cuauhtémoc, 06720 Ciudad de México, CDMX, Mexico City, Mexico; 2 Instituto Nacional de Ciencias Médicas y Nutrición Salvador Zubirán, Vasco de Quiroga 15, Belisario Dominguez Sección XVI, 14080 Mexico City, PC Mexico

**Keywords:** Kidney cancer, Tyrosine kinase inhibitor, Sunitinib, Sorafenib, Clear cell carcinoma, Cohort, Renal cell carcinoma, VEGF

## Abstract

**Background:**

Sequential inhibition of the vascular endothelial growth factor (VEGF) pathway with sorafenib could be useful for patients with metastatic renal cell carcinoma (RCC). Our aim was to determine the activity and tolerability of sorafenib as a second-line therapy in advanced RCC initially treated with a different VEGF-tyrosine kinase inhibitor (TKI).

**Methods:**

A prospective observational cohort in Mexico (2012–2019). We included 132 subjects with metastatic RCC and who had progression despite treatment with sunitinib. The primary end-point was time to disease progression as evaluated every 12–16 weeks.

**Results:**

The mean age of the cohort was 59 years (interquartile range [IQR] 50–72), 96 (73%) were men, and 48 (36%) had a favorable prognosis according to the IMDC (International Metastatic RCC Database Consortium) prognostic model. The median progression-free survival (PFS) and overall-survival after the introduction of sorafenib treatment was 8.6 months (95% confidence interval [CI]: 6.7–10.5) and 40 months (95% CI: 34.5–45.4) respectively. The median overall survival from RCC diagnosis to death was 71 months (95% CI: 58.2–83.8). On multivariable analyses, age > 65 years was associated with a longer PFS (HR 0.51; 95% CI: 0.31–0.86; *p* = 0.018). The median PFS in subjects aged > 65 years was longer compared to subjects ≤65 years (14.0 [95% CI: 9.2–18.8] vs. 7.2 months [95% CI: 5.3–9.1]; *p* = 0.012). Adverse events grade ≥ 3 associated with sorafenib occurred in 38 (29%) patients.

**Conclusion:**

Sequential inhibition of VEGF with sorafenib as a second-line treatment may benefit patients with metastatic RCC, especially in subjects > 65 years old.

**Supplementary Information:**

The online version contains supplementary material available at 10.1186/s12885-020-07720-5.

## Background

Renal cell carcinoma (RCC) is one of the most common types of cancer and its incidence has been rising by approximately 0.6% each year; however, death rates have been falling by 0.7% each year [1, 2]. Patients with advanced RCC develop new metastatic lesions up to 10–30% despite being treated with new drugs, including vascular endothelial growth factor-tyrosine kinase inhibitor (VEGF-TKI) or targeted therapies [3, 4]. Additionally, between 20 to 30% of patients with localized RCC experience relapse in distant sites within 3 years of surgical resection [5–8].

Sorafenib tosylate is a non-selective VEGF-TKI that suppresses multiple isoforms of the intracellular serine/threonine kinase, including the VEGF receptors type 1, 2, and 3 [9, 10]. Sorafenib has been tested as a second-line therapy, especially in patients with RCC initially treated with cytokine therapy [11, 12]. In patients with advanced RCC treated initially with sunitinib, the sequential use of a second drug with a similar molecular target could raise doubts about its clinical usefulness [13]. Nevertheless, there are differences in target specificities among the TKIs demonstrated in pharmacological research [14]. Retrospective observational studies have shown a lack of cross resistance between sequential use of TKIs and the distinctive toxicity spectra that occasionally permit tolerance of one TKI over another [15].

The National Comprehensive Cancer Network (NCCN) recommended sorafenib for patients whose disease progressed on a prior therapy as useful only under certain circumstances (category 2A), given the lack of clinical studies with sorafenib as a second-line therapy and the existence of other alternative and effective second-line therapeutic options tested in clinical trials (e.g., cabozantinib, nivolumab, or ipilimumab/nivolumab) [16]. Nevertheless, sorafenib remains the main second-line therapy prescribed in many developing countries because of its availability, relative low-cost, favorable clinical efficacy, and safety. Our aim was to determine the progression-free survival (PFF) of sorafenib as a second-line sequential TKI therapy in advanced RCC initially treated with sunitinib. In addition, as secondary end points, it was determined overall survival (OS) and drug toxicity.

## Methods

This observational cohort study was performed at one tertiary-care center in Mexico City (Centro Médico Nacional Siglo XXI), which belongs to the largest public social security institution in the country. It is a national reference center for specialized treatment of metastatic disease. From July 2012 to July 2019, we included subjects aged ≥18 years with RCC biopsy-proven, who had experienced RCC disease progression after initial treatment with sunitinib and nephrectomy. In our center, 2nd line treatments such as cabozantinib, nivolumab, ipilimumab, or axitinib are not available most of the time and sorafenib is the treatment available as second line. The study was approved by the institutional review board (reference number R-2012/2019–3602-007). Patients´ written informed consent was exempted because of the observational nature of the study.

All subjects received sorafenib 400 mg orally twice a day on a continuous dosing schedule until disease progression or intolerable toxicity. Dose reductions, delays, or temporary interruptions of sorafenib were assessed prospectively independently by one evaluator. All clinical evaluations were performed every 4 weeks. Evaluation included a clinical interview, a physical examination, and a comprehensive metabolic panel. Imaging with computed tomography or magnetic resonance imaging was performed every 12–16 weeks and was scored per RECIST V.1.1 criteria by an expert radiologist [17]. PFS with sorafenib was defined as the time from the start of the sorafenib treatment to disease progression as the primary end-point. Subjects who did not experience progression after treatment suspension for any cause or who were lost during follow-up were censored. Risk factors were classified according to the International Metastatic Renal Cell Carcinoma Database Consortium (IMDC) prognostic model [18]. Strictly regular monitoring of hypothyroidism was performed on a regular basis every 3–6 months. Echocardiography in asymptomatic patients was not included in the active surveillance protocol. We initially calculated a minimal sample size based of 110 patients based on the calculation methods for one sample non-parametric survival test/confidence interval [19] assuming a time at analysis of 12 months, a null survival probability of 0.4, a sorafenib survival probability of 0.55, a follow-up time of 24 months, α value of 0.05, and 1-β of 0.8.

Descriptive quantitative results are presented as mean ± standard deviation for normally distributed data or median (interquartile range [IQR]) for nonnormally distributed data; *t*-test or nonparametric Mann–Whitney *U* test were used to investigate differences. Cross-tabulated data were analyzed with chi-square or Fisher tests. Kaplan–Meier analyses were employed to summarize time-to-event data and statistical differences were estimated by the log rank test and Cox proportional hazard model. Cox proportional hazard regression models were performed to determined variables that were associated with risk of death. Variables with *p* < 0.15 in the univariate analysis were included in the multivariate models. Logistic regression analysis was performed to assess the risk factors associated with toxicity risk. We compared the agreement between toxicity grade reports related to sunitinib and sorafenib therapies using a concordance test (Kendall’s tau-b). Subjects with missing data were excluded from analysis. All tests of significance were two-tailed and differences were considered statistically significant at a *p*-value < 0.05. All statistical analyses were performed using SPSS software (v. 21.0; IBM SPSS, Armonk, NY, USA) and graphics were analyzed using GraphPad Prism 5 (GraphPad Software, San Diego, CA, USA).

## Results

We included 132 subjects with metastatic RCC treated by surgical resection and treated with sunitinib as the first-line treatment (Supplementary Fig. 1 includes flow chart of patients who met inclusion/exclusion criteria for the cohort study). Their baseline demographic and clinical data are described in Table 1. The mean age of the cohort was 59 (IQR 50–72 years, 96 (73%) were men, 48 (36%) had a favorable prognosis according to the IMDC criteria risk factors, and the most common sites of metastasis were lung (*n* = 70, 53%) and bone (*n* = 17, 23%). Eighty-five (64%) subjects were treated initially only with surgical excision and progression was diagnosed after 6 months of active surveillance. The remaining subjects (*n* = 47, 46%) received TKI as first-line therapy within 3 months of their RCC diagnosis. All subjects received a sunitinib (median dose of 37.5 mg/day). Interferon treatment was employed before TKI treatment in only two cases. The median time between first-line therapy (sunitinib) and second-line therapy (sorafenib) was 12.7 months (IQR, 13–38). A good Karnofsky performance status scale score ≥ 80 was observed in 132 subjects (100%). Forty-four (33%) subjects had ≥2 metastatic sites at the start of their sorafenib treatment. The median follow-up of the entire cohort after sorafenib treatment was 7 months (range 2 to 61 months).

**Table 1:** Baseline clinical characteristics before sorafenib treatment and response rates after treatment

Characteristics before sorafenib treatment (*n* = 132)	Values*
Age, years	59 (50–72)
Male, n (%)	96 (73)
Age > 60 years, n (%)	59 (45)
Nephrectomy, n (%)	132 (100)
Stage at RCC diagnosis, n (%)	
Unknown	32 (24)
1	4 (3)
2	11 (8)
3	32 (24)
4	53 (41)
Karnofsky performance status scores	
100	1 (1)
90	82 (62)
80	49 (37)
High blood pressure, n (%)	44 (33)
Hypothyroidism, n (%)	8 (6)
Site of metastasis	
Lung, n (%)	70 (53)
Bone, n (%)	17 (13)
Liver, n (%)	18 (14)
Lymph node, n (%)	12 (9)
First-line and concomitant treatments	
Sunitinib, n (%)	132 (100)
Interferon, n (%)	3 (2)
Radiotherapy, n (%)	27 (20)
Zoledronic acid, n (%)	2 (2)
Risk status, % (n)**	
Favorable risk	48 (36)
Intermediate risk	81 (61)
High risk	3 (23)
Hemoglobin, g/dL	13.9 ± 1.8
Leucocytes, 10^9^/L	6.5 (5.3–8.2)
Neutrophils, 10^9^/L	3.9 (2.6–5.0)
Platelets, 10^9^/L	235 (186–310)
Albumin-adj calcium, g/dL	9.5 (9.0–9.8)
LDH, mg/dL	178 (151–234)
**Response rates after sorafenib treatment**	
Complete Response, n (%)	3 (2)
Partial Response, n (%)	11 (8)
Stable Disease, n (%)	13 (10)
Progressive Disease, n (%)	105 (80)

* Continuous variables are expressed as mean ± SD or as median (25th–75th percentile), categorical variables are expressed as n (%). **Risk status was classified according to the International Metastatic Renal Cell Carcinoma Database Consortium (IMDC model), in which favorable risk is equivalent to no factors, intermediate risk, 1–2 factors, and high risk, 3–6 factors [18]. *LDH* Lactate dehydrogenase, *Albumin-adj* Albumin-adjusted

**Fig. 1:**
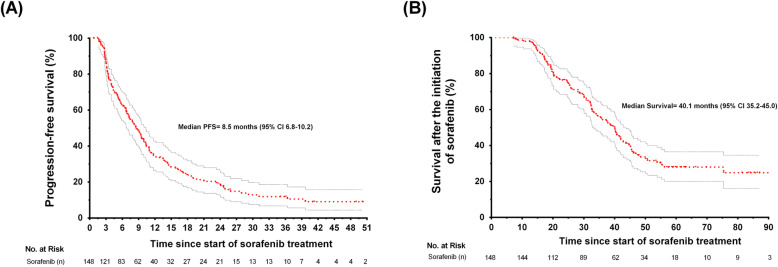
Progression-free survival (**a**) and survival (**b**) after the introduction of sorafenib treatment

The median PFS and survival after the introduction of sorafenib treatment was 8.6 (95% confidence interval [CI]: 6.7–10.5) and 40 months (95% CI: 34.5–45.4), respectively (Fig. 1). OS since cancer diagnosis was 71 months (95% CI: 58.2–83.8). The sum of PFS of first-line sunitinib and second-line sorafenib was a median time of 29.7 months (IQR, 23.7–38.0). Progressive disease after sorafenib treatment occurred in 105 (80%) subjects. The common sites of second progression were lung in 27 (20%), bone in 29 (22%), and central nervous system in 9 (7%) subjects. At the end of follow-up, 87 (66%) had died. Complete response, partial response, and stable disease were observed in 3 (2%), 11 (8%), and 13 (12%) subjects respectively. At the time of analysis, 30 (23%) of all patients were still alive and 15 (10%) were lost to follow-up. Third-line treatments for progressors were interferon (26/105, 25%), radiotherapy (23/105, 22%), and nivolumab (1/105, 1%). The median PFS was longer during the first TKI treatment (sunitinib) compared to the second TKI treatment with sorafenib (16.0 [95% CI: 13.5–17.7] vs. 8.6 months [95% CI: 6.7–10.5]; *p* < 0.001; hazard ratio = 1.55; 95% CI: 1.18–2.03; Supplementary Fig. 2).

**Fig. 2:**
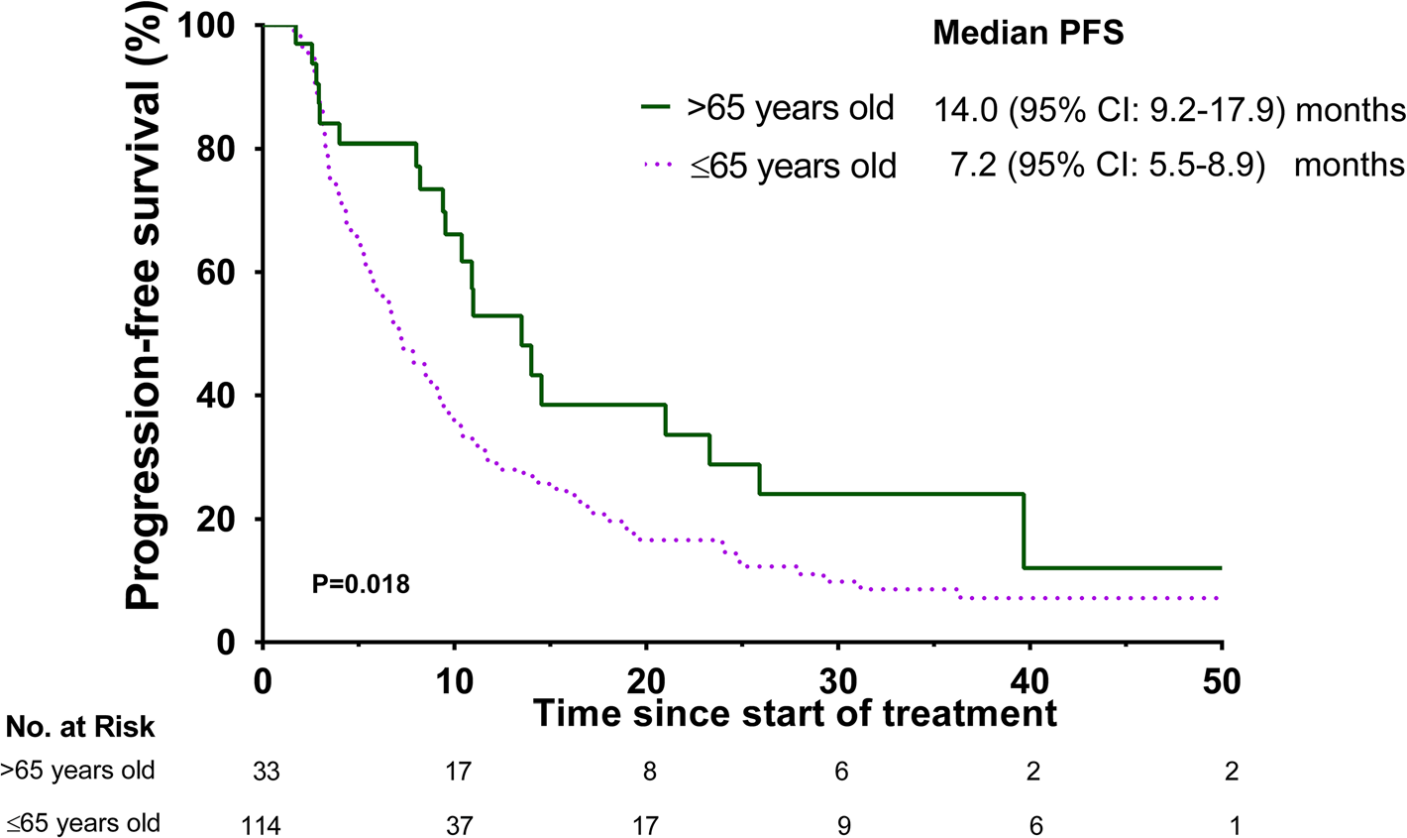
Progression-free survival on sorafenib in subjects > 65 and ≤ 65 years old

We performed analyses of several clinical parameters to identify those variables associated with RCC progression. We identified age as the only significant variable differing between subjects who progress on sorafenib treatment versus nonprogressors (Table 2 and Table 3). Median PFS in subjects > 65 years old was longer (14.0 months, 95% CI: 9.2–18.8) compared to subjects ≤65 years old (7.2 months, 95% CI: 5.3–9.1 months; *p* = 0.012; Fig. 2). Age > 65 years independently decreased the odds of RCC progression after sorafenib treatment (hazard ratio [HR] = 0.51; 95% CI: 0.31–0.86; *p* = 0.018). We observed a non-statistically significant difference in the survival after the introduction of sorafenib in subjects > 65 years old compared with subjects ≤65 years old (43 [95% CI: 30–40] vs. 36 months [95% CI: 40–45]; *p* = 0.06). The median OS was 69 months (95% CI: 52–85) in subjects aged ≤65 years compared to 106 months (95% CI 66–145) in those > 65 years old (*p* = 0.091). Age > 65 years had a non-statistically significant HR of 0.62 (95% CI: 0.36–1.08, *p* = 0.09) for OS. Prognosis according to the IMDC prognostic model was not different between progressors and nonprogressors, the median PFS was 9.4 [95% CI: 5.6–13.3] in metastatic RCC with no IMDC risk factor versus 8.5 months [95% CI: 6.0–11.0] in subjects with ≥1 IMDC risk factors (*p* = 0.08; Supplementary Fig. 3).

**Table 2:** Characteristics of subjects with advanced RCC with progressors versus nonprogressors on sorafenib treatment

Baseline characteristics*	Progressors,*n* = 105 (80%)	Nonprogressors,*n* = 27 (20%)	*P*
Age, years	58 (49–64)	63 (55–73)	0.004
Age > 65 years, n (%)	17 (16)	11 (42)	0.007
Male, n (%)	76 (72)	20 (74)	1.00
Favorable prognosis**, n (%)	37 (35)	11 (40)	0.51
Radiation therapy, n (%)	22 (21)	5 (19)	1.00
High blood pressure, n (%)	35 (33)	9 (35)	1.00
Karnofsky performance status ≥90, n (%)	66 (63)	16 (59)	1.00
≥ 2 metastatic sites, n (%)	38 (36)	6 (22)	0.25
Hemoglobin, g/dL	13.9 ± 1.9	13.7 ± 1.4	0.52
Leucocytes, 10^9^/L	6.3 (5.2–7.9)	7.1 (5.5–8.9)	0.36
Neutrophils, 10^9^/L	3.7 (2.6–4.8)	4.2 (2.7–5.1)	0.34
Platelets, 10^9^/L	235 (187–311)	244 (174–294)	0.70
Albumin-adj calcium, mg/dL	9.5 (9.0–9.9)	9.5 (9.2–9.8)	0.78
LDH, mg/dL	176 (150–227)	180 (162–287)	0.33

* Continuous variables are expressed as mean ± SD or as median (25th–75th percentile), categorical variables are expressed as n (%). ** Favorable group was classified according to the International Metastatic Renal Cell Carcinoma Database Consortium (IMDC model), in which favorable risk is equivalent to no factors. *TKI* Tyrosine kinase inhibitor, *LDH* Lactate dehydrogenase, *Albumin-adj* Albumin-adjusted

**Table 3:** Multivariate Cox proportional hazard models for the predictors of progression free-survival

Factors	Univariate PFS		Multivariate PFS	
	HR (95% CI)	*P*	HR (95% CI)	*P*
Age > 65, yes	0.51 (0.31–0.87)	0.013	0.52 (0.31–0.88)	0.014
Male, yes	1.10 (0.72–1.69)	0.66	1.02 (0.65–1.56)	0.95
Favorable prognosis, yes	1.43 (0.95–2.14)	0.084	1.41 (0.93–2.1)	0.099
Karnofsky performance status ≥90, n (%)	1.18 (0.79–1.76)	0.43	–	–
≥ 2 metastatic sites, n (%)	1.35 (0.90–2.01)	0.15	–	–
Hemoglobin, g/dL	0.98 (0.87–1.10)	0.69	–	–
Leucocytes, 10^9^/L	1.03 (0.92–1.15)	0.59	–	–
Neutrophils, 10^9^/L	1.03 (0.91–1.17)	0.65	–	–
Platelets, 10^9^/L	1.00 (1.00–1.005)	0.044	–	–
Total calcium, mg/dL	1.074 (0.83–1.38)	0.58	–	–
LDH, mg/dL	0.99 (0.99–1.00)	0.28	–	–

Abbreviations: *PFS* Progression free-survival, *HR* Hazard ratio, *CI* Confidence interval. Favorable group was classified according to the International Metastatic Renal Cell Carcinoma Database Consortium (IMDC model), in which favorable risk is equivalent to no factors

Adverse events associated with sorafenib occurred in 123 (93%) subjects and included hand-foot syndrome (*n* = 105, 80%), diarrhea (*n* = 100, 76%), hypothyroidism (*n* = 28, 29%), and mucositis (*n* = 70, 53%) (Table 4). Any adverse events corresponding to a grade ≥ 3 occurred in 38 (29%) patients. In 66 (50%) cases, the sorafenib dose was adjusted at first visit because of toxicity effects. Subjects who developed hypothyroidism related to sorafenib had higher baseline levels of thyroid-stimulating hormone compared to those who did not develop any thyroid disorder (2.9 ± 0.9 vs. 2.0 ± 1.2 mU/mL; *p* = 0.045). Development of hypothyroidism during sorafenib therapy was not associated with a favorable response as defined as complete response, partial response, or stable progression (OR = 0.83; 95% CI: 0.3–2.7; *p* = 0.76). Three subjects treated with sorafenib discontinued therapy due to intolerance (hepatotoxicity, ischemic cardiomyopathy, and severe high blood pressure). There was no concordance between the severity of drug toxicity reports related to sunitinib and sorafenib treatments (Kendall’s tau-b = 0.016; *p* = 0.92). Likewise, there were no associations for type of adverse events related to sunitinib and sorafenib therapies, except for nausea, which had a slight concordance between sorafenib and sunitinib use (Kendall’s tau-b = 0.21; *p* = 0.015). Sixteen subjects (12%) with adverse events grade ≥ 3 related to sorafenib did not have any history of previous adverse events grade ≥ 3 during sunitinib therapy. Use of sorafenib in patients with history of adverse events grade ≥ 3 related to sunitinib was not associated with toxicity risk grade ≥ 3 after sorafenib therapy (OR = 1.08; 95% CI: 0.53–2.23; *p* = 0.83).

**Table 4:** Common adverse events of sequential TKI treatment

Adverse events related to first-line treatment with sunitinib	Any grade (%)	Grade 1 (%)	Grade 2 (%)	Grade 3 (%)	Grade 4 (%) **
Any adverse event*	129 (97)	20 (15)	57 (43)	51 (38)	1 (1)
Thrombocytopenia	39 (30)	8 (6)	13 (10)	17 (13)	1 (1)
Neutropenia	16 (12)	7 (5)	3 (2)	6 (5)	
HFSR**	104 (79)	53 (40)	37 (28)	14 (11)	
Mucositis	109 (83)	43 (33)	42 (32)	24 (18)	
Diarrhea	89 (67)	51 (39)	26 (20)	12 (8)	
Nausea	25 (19)	20 (15)	5 (4)	0 (0)	
Fatigue	79 (60)	51 (39)	22 (17)	6 (4)	
High blood pressure	37 (28)	23 (17)	10 (8)	4 (3)	0 (0)
Adverse events related to second-line treatment with sorafenib					
Any adverse event	123 (93)	22 (17)	25 (49)	37 (28)	1 (1)
Hypothyroidism	38 (29)	25 (18)	12 (9)	1 (1)	–
HFSR	105 (80)	56 (42)	30 (23)	19 (14)	
Rash	26 (20)	19 (14)	5 (4)	2 (2)	
Mucositis	70 (53)	44 (33)	20 (15)	6 (5)	
Diarrhea	100 (76)	51 (39)	43 (33)	6 (4)	
Nausea	44 (34)	38 (29)	5 (4)	1 (1)	
Fatigue	106 (80)	62 (47)	35 (27)	9 (7)	
High blood pressure	12 (9)	4 (3)	7 (5)	0 (0)	1 (1)

*Any adverse event for patient, graded as maximum; **HFSR Hand-foot skin reaction; **Grade 4 only in those cases classified according to toxicity grades by the common Terminology Criteria for Adverse Events (CTCAE) classification [20]

## Discussion

In this prospective cohort study of 132 Mexican patients with metastatic RCC treated initially with a TKI as first-line therapy, we observed that sequential TKI therapy with sorafenib as second-line therapy is an acceptable treatment option given the outcomes observed: i.e., a median PFS of almost 9 months and a median survival after the introduction of sorafenib of more than 40 months. In addition, sorafenib administration was safe considering sorafenib-related adverse events.

It is difficult to compare PFS obtained from second-line treatments across observational cohorts and clinical trials, given the variations in the designs, patient characteristics, response criteria, and definitions employed among studies. Nevertheless, some general aspects could be inferred. The median PFS with sorafenib in our cohort seems to be similar to the median PFS of 4 to 7 months obtained in other clinical trials for advanced RCC with everolimus, axitinib, nivolumab, cabozantininb or nivolumab/ipilimumab, [21–25]. Only lenvatinib/everolimus (14.6 months) [26] had shown a median PFS longer than other second-line therapies in randomized clinical trials with a higher rate of related adverse events. According to our results, we propose that sorafenib could be considered as a feasible option for advanced RCC in certain clinical scenarios, considering patient preferences, specific comorbidities [27], tolerability, and availability.

We need to understand the clinical benefits of actual treatments to obtain fair comparisons with new agents for second-line therapies, especially in minority populations and elderly patients [3]. NCCN guidelines suggest that for subsequent therapy in metastatic RCC, the simplest approach is to change the mechanism of action related to the second-line therapy, e.g., if a subject was treated with a TKI as first-line treatment, a PD-1 agent should be the second option. Nevertheless, observational data support the use of sequential TKIs following the treatment with an initially different TKI [2, 28]. Treatment of metastatic RCC with two TKIs in sequence, both sharing a similar molecular target yet with different clinical effects, could be comparable to newer, more-expensive agents, which are mostly unavailable in developing countries.

In our cohort, it is possible that the majority of patients showed an acceptable PFS time on sorafenib therapy, which is explained by the favorable risk prognosis when the second-line therapy began. Other studies had shown lower PFS with sorafenib. For example, in the AXIS clinical trial, the median PFS with sorafenib as second-line therapy was 4.7 months, where only 28% had a favorable classification according to the Memorial Sloan-Kettering Cancer Center risk [22]. Nevertheless, the intermediate-prognosis group in our study had a median progression time of more than 7 months, which seems to be superior to the AXIS trial results. We believe that our patients were highly selected, and our results should not be over-interpreted.

Despite clinically improved outcomes in advanced RCC, it is believed that resistance to VEGF-targeted treatment develops in nearly all patients with RCC [5]. In our study, the occurrence of cross-resistance was not observed in all cases during follow-up. Nearly 21% of subjects did not show absolute cross-resistance between the two sequential TKIs (sunitinib and sorafenib). Multiple studies have shown that second-line sorafenib after sunitinib progression is well tolerated and safe over the long term [29, 30]. These findings show the urgent need to investigate and understand the acquired resistance to TKIs in patients with RCC.

In a retrospective study with 33 patients who had experienced RCC progression treated with sequential use of either sorafenib or sunitinib, Calvani et al. observed that survival on second-line TKI was longer in the patients who received sorafenib first compared to those treated with sunitinib first (median PFS = 11 vs. 3 months). In our results, the increase in median PFS with sunitinib was longer (15 months) than second-line treatment with sorafenib (8.5 months) and the total PFS (the sum of PFS of first-line sunitinib and second-line sorafenib) was longer compared to the referred study (29.8 vs. 10 months, respectively) [31].

We found a high rate of sorafenib-related adverse effects (93%), although we did not regularly perform echocardiography in all patients. According to other studies, sorafenib could be associated with almost 100% of adverse effects [32–34]. These high rates of toxicity could be attributed to differences in methods to report adverse events. In our study, sorafenib treatment was associated with an astounding number of mucocutaneous side effects, especially hand-foot skin disease compared to reports from other clinical studies [22, 31, 35]. In an Asian population, fatigue and hand-foot skin reactions were more common compared to diarrhea, which is the most common adverse effect in non-Asian populations [36]. In clinical trials with predominantly non-Asian or non-Latin-American patients, hand-foot skin adverse effects has been observed in between 27 to 30% of patients [22, 23].

Our study limitations are related to its observational nature, which could be subject to bias, the absence of a comparative treatment, and the inclusion of subjects treated in only one center. In addition, we did not known if no treatment at all could be similar to sorafenib as a second treatment line in many patients. The absence of a comparison group prevents us from making inferences about the real usefulness of sorafenib. In our study, only 14/132 (11%) of subjects had complete and/or partial response, which is a very low proportion of success which is consistent with prior studies of second line treatment with sorafenib after failure of first line sunitinib [29, 30]. Nevertheless, in our center, we have limited access to alternative second line therapies (e.g. immune checkpoint inhibitors) which is a common problem in developing countries. Our data from real clinical scenarios could help to improve decision making in RCC patients after failure of first line sunitinib.

## Conclusion

In this observational cohort study of sorafenib as a second-line therapy in patients with advanced RCC, we observed a median PFS of 8.6 months, a response rate of 10%, and a 29% of serious adverse events related to sorafenib therapy. In subjects > 65 years old, sequential inhibition of VEGF with sorafenib as a second-line treatment may be an option when other treatments are not available. Further large clinical trials including sorafenib as a comparator versus new agents are needed.

## Supplementary Information

**Additional file 1: Supplementary Fig. 1.** Cohort flow chart illustrating the inclusion and exclusion of RCC subjects in the study. **Supplementary Fig. 2.** Progression-free survival on first-line tyrosine kinase inhibitor (sunitinib) versus second-line treatment with sorafenib. **Supplementary Fig. 3.** Progression-free survival on sorafenib in subjects with no risk factors (favorable prognosis) and ≥ 1 risk factors (intermediate prognosis).

## Data Availability

The data that support the findings of this study are available on request from the corresponding author, J.C.R.S. Also, the data are publicly available at https://www.researchgate.net/publication/343294988_sorafenib_analysis_2020 with the following identifier: DOI: 10.13140/RG.2.2.28339.04647.

## Abbreviations

CI: Confidence interval
IMDC: International metastatic RCC database consortium
IQR: Interquartile range
NCCN: National comprehensive cancer network
OS: Overall survival
PFS: Progression-free survival
RCC: Renal cell carcinoma
TKI: Tyrosine kinase inhibitor
VEGF: Vascular endothelial growth factor
