# Supplementary material for: Sorafenib as a second-line treatment in metastatic renal cell carcinoma in Mexico: a prospective cohort study
Source: BMC Cancer. 2021 Jan 5;21:16. doi: 10.1186/s12885-020-07720-5 (PMC7786959; doi:10.1186/s12885-020-07720-5)
Supplement: Supplementary file 1 — Additional file 1: Supplementary Fig. 1. Cohort flow chart illustrating the inclusion and exclusion of RCC subjects in the study. Supplementary Fig. 2. Progression-free survival on first-line tyrosine kinase inhibitor (sunitinib) versus second-line treatment with sorafenib. Supplementary Fig. 3. Progression-free survival on sorafenib in subjects with no risk factors (favorable prognosis) and ≥ 1 risk factors (intermediate prognosis). [file 12885_2020_7720_MOESM1_ESM.docx]

**Supplementary figure 1.**

Cohort flow chart illustrating the inclusion and exclusion of RCC subjects in the study.

Of 148 participants assessed for study eligibility, 132 were included. Reasons for screening

failures are displayed in figure.


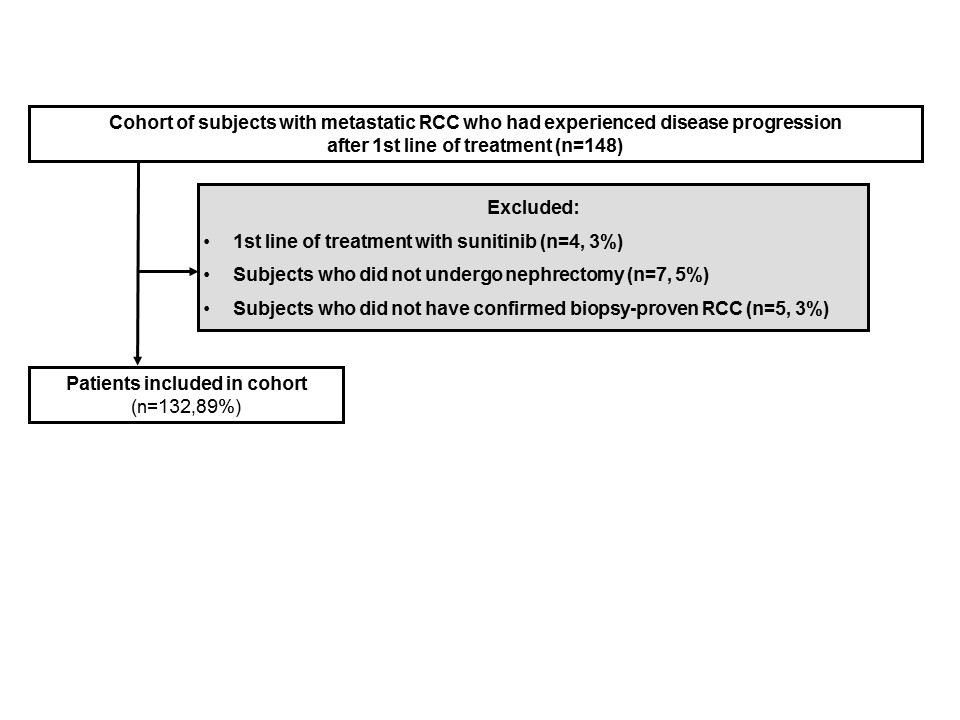


**Supplementary figure 2** Progression-free survival on first-line tyrosine kinase inhibitor (sunitinib) versus second-line treatment with sorafenib





**Supplementary figure 3.** Progression-free survival on sorafenib in subjects with no risk factors (favorable prognosis) and ≥1 risk factors (intermediate prognosis)
